# Journal De La Physiologie; Dr. Brown-Séquard

**Published:** 1860-05

**Authors:** 


— The January number of the "*Journal de Physioligie*" has been received, and will be noticed in our next number. The removal of its editor, Dr. Brown-Séquard, to London, will not at all interfere with the continuance of this journal. It will be published, as heretofore, at Paris. Dr. Brown-Séquard has been appointed to the charge of a new hospital at London, called the "National Hospital for the Paralyzed and Epileptic." Clinical lectures by this distinguished physiologist will soon be commenced at the hospital.

